# 

**Published:** 1810-01

**Authors:** 


### MEDICAL

AND

### PHYSICAL

### JOURNAL.

CONDUCTED BY

T. BRADLEY, M. D.

AND

W. SHEARMAN, M. D.

VOL. XXIII.

FROM JANUARY TO JUNE, 1810.

*LONDON.*

PRINTED FOR RICHARD PHILLIPS, NO. 6, BRIDGE-STREET, BLACKFRIARS.

BY WILLIAM THORNE, RED LION COURT, FLEET STREET.

### NEW MEDICAL PUBLICATIONS.

Two Engravings; one representing the Basis of the Human Brain, the other the Cavity in which it is contained, accompanied with two Outline Plates, and Figures of Reference. By T. J. Pettigrew, royal 4to. 1l. 1s.

Letters concerning the Diseases of the Urethra. By Charles Bell, 8vo. 7s. 6d.

Cases and Observations in Surgery. By Walter Weldon, 3s. 6d.

An Inquiry into the Seat and Nature of Fever. By Henry Clutterbuck, M. D. Part I. 8vo. 9s.

A Treatise on the Venereal Disease, by John Hunter, with an Introduction and Commentary, by Joseph Adams, M. D. Author of Observations on Morbid Poisons, &c. 8vo, with Plates, 14s.

---

### CORREPONDENCE.

We are truly concerned that our ingenious and witty Correspondent from Nottinghamshire, should have supposed any of our Remarks on Reviewing could refer to him.

Mr. Earle's valuable communication was received too late to complete the engraving for the present month. It shall appear in our next.

